# Solitary colorectal liver metastasis: overview of treatment strategies and role of prognostic factors

**DOI:** 10.1007/s00432-021-03880-4

**Published:** 2021-12-16

**Authors:** S. Acciuffi, F. Meyer, A. Bauschke, R. Croner, U. Settmacher, A. Altendorf-Hofmann

**Affiliations:** 1 Department of General, Abdominal and Vascular Surgery, University Hospital, Leipziger Str. 44, 39120 Magdeburg, Germany; 2 Department of General, Abdominal and Vascular Surgery, University Hospital, Am Klinikum 1, 07747 Jena, Germany

**Keywords:** Solitary colorectal liver metastasis, Liver surgery, Local ablation

## Abstract

The following is an overview of the treatment strategies and the prognostic factors to consider in the therapeutic choice of patients characterized by solitary colorectal liver metastasis. Liver resection is the only potential curative option; nevertheless, only 25% of the patients are considered to be eligible for surgery. To expand the potentially resectable pool of patients, surgeons developed multidisciplinary techniques like portal vein embolization, two-stage hepatectomy or associating liver partition and portal vein ligation for staged hepatectomy. Moreover, mini-invasive surgery is gaining support, since it offers lower post-operative complication rates and shorter hospital stay with no differences in long-term outcomes. In case of unresectable disease, various techniques of local ablation have been developed. Radiofrequency ablation is the most commonly used form of thermal ablation: it is widely used for unresectable patients and is trying to find its role in patients with small resectable metastasis. The identification of prognostic factors is crucial in the choice of the treatment strategy. Previous works that focused on patients with solitary colorectal liver metastasis obtained trustable negative predictive factors such as presence of lymph-node metastasis in the primary tumour, synchronous metastasis, R status, right-sided primary colon tumor, and additional presence of extrahepatic tumour lesion. Even the time factor could turn into a predictor of tumour biology as well as further clinical course, and could be helpful to discern patients with worse prognosis.

## Introduction

Colorectal cancer is the third most common tumor in men and the second in women, with an estimate of 1.8 million new cases and approximately 881,000 deaths in 2018 (Bray et al. 2018). Hepatic metastases develop in approximately 50% of colorectal cancer cases (Kanas et al. 2012). The liver, in addition to being the most common site of metastases, is also the first and only area of spread in 30–40% of patients (Hadden et al. 2016).

Surgery is considered the gold standard treatment and the only potentially curative option for colorectal liver metastases (CLM). Unfortunately, despite the oncological and surgical advances made, only about 25% of patients affected are suitable for resection (Engstrand et al. 2018). The development of surgical techniques, the increasing efficacy of modern chemotherapy with or without biological agents, and the emergence of multidisciplinary approaches have allowed patients with conventionally unresectable CLM to undergo surgery (Imai et al. 2019).

The decision of the treatment strategy should be based on patients’ features and on trustable as well as relevant predictive factors to obtain the best oncologic result with as few complications as possible. The following is an overview of the treatment strategies and the prognostic factors to consider in the therapeutic choice of patients characterized by, in particular, solitary liver metastasis (SLM), a homogeneous group of patients, with—therefore—reliable statements on factors and parameters relevant for early post-operative and long-term oncosurgical outcome.

## Corner points

### Surgical treatment

In the late 1990s, surgery was offered only to a high selected group of patients with liver-limited disease, confined to only one lobe. These patients should have no more than three metastasis and the larger one should not be bigger than 5 cm. Moreover, the resection had to be technically feasible with at least 1 cm tumour-free margins (Kanas et al. 2012). Following these criteria for about 90% of the patients, the disease was considered unresectable and a 5 year survival of merely 3.3% and 6.1% for synchronous and metachronous metastasis, respectively, was reported (Manfredi et al. 2006). Later on, it became clear that the first option to improve overall survival (OS) was to expand surgery indications.

To date, every patient with a disease control is a candidate for surgery. Independently of tumor burden, the most important factor that limits the surgeon is the future liver remnant that should be at least 30% or a remnant liver-to-body weight ratio > 0.5 (E Van Cutsem et al. 2016a, b).

The presence of limited extrahepatic disease is no longer considered an absolute contraindication. The concept of oligometastases has gained importance in colorectal cancer, since it has been demonstrated that patients who underwent resection of hepatic and extrahepatic (lung or peritoneum or lymph node) metastasis had survival data exceeding the best outcomes in patients receiving systemic chemotherapy alone (Hadden et al. 2016). Oligometastases are characterized by a few lesions (generally up to 5) localized to a few sites (up to 2 or occasionally 3), and are associated with the possibility of achieving complete ablation of all tumour masses, using surgical R0 resection and/or local ablation strategies, either initially or possibly after induction treatment with systemic therapy (Eric Van Cutsem et al. 2016a, b). For these patients, a potentially curative approach exists.

-In patients with synchronous resectable metastases, there are three operative options including,
-Staged resection with colon first strategy,
-Staged resection with liver-first strategy, or
-Simultaneous resection of both primary tumour and metastases.

In case of risk of primary tumour complications such as bleeding, obstruction, or perforation, the colorectal-first approach should be favored. The liver-first sequence is most suited to rectal cancers, so that the liver metastases are not left untreated during the radiation portion of treatment to the rectum. The priority in staged resections may be given to colorectal-first or liver-first strategies depending on possible complications related to the primary tumour or on the progression of CLM during the treatment of the primary tumour (Abdalla et al. 2013). A recent study proposed a tumor burden-driven strategy: the liver-first approach has a clear survival advantage in patients with multiple bilobar metastases. For patients with solitary liver metastases or multiple unilobal metastasis, the staged procedures (liver first vs primary first) showed to be equivalent. Simultaneous resections should be cautiously considered because of increased mortality risk whenever a major hepatectomy is needed (Giuliante et al. 2021).

It has been evaluated if the use of neoadjuvant chemotherapy before liver surgery could improve the oncological outcome. Chemotherapy addresses micrometastases and circulating tumor cells to reduce recurrent metastatic disease in resectable CLM, and downsizes liver lesions with the aim of achieving resectability in initially unresectable CLM. On the other hand, neoadjuvant treatment strategies with cytotoxic doublets and targeted agents cause time-dependent liver damage and steatohepatitis resulting in increased liver failure after surgery (Aigner et al. 2017). The only controlled, phase-III trial that compared perioperative therapy with FOLFOX (before and after surgery) with surgery alone for patients with liver-only metastases showed that perioperative chemotherapy increases progression-free survival, but there was no statistically significant survival difference between the two groups (Nordlinger et al. 2008, 2013).

The decision of which therapy strategy should be preferred must be discussed and made within a multidisciplinary board. Furthermore, the potential resectability of the metastasis should always undergo the evaluation of an expert liver surgeon to not miss the opportunity of the resection in cases of big or central located metastasis. It is conventionally accepted that resection of up to 75% of the total liver volume or six liver segments with an adequate inflow and outflow can be safely performed in patients with normal liver parenchyma. In the setting of steatosis, steatohepatitis, or cirrhosis, the volume of liver that can safely be resected may have to be dramatically reduced to avoid the risk of post-operative hepatic failure. A work of Truant et al. demonstrated that a remnant liver volume-to-body weight ratio ≤ 0.5% is a trustable tool to predict post-operative hepatic liver failure and post-operative mortality (Truant et al. 2015). In an attempt to improve the functional liver remnant, various techniques have been employed including portal vein embolization (PVE), two-stage hepatectomy, or portal vein ligation. In the portal vein embolization, after selective catheterization, one of two portal branches and its ramifications are occluded through the diffusion of embolizing agent. Two-to-four weeks after PVE, patients received a contrast-enhanced liver CT or liver MRI to assess hypertrophy of the future liver remnant. A recent retrospective study demonstrated that the presence of metastases in the future liver remnant before PVE had no impact on progression-free or OS, which supports aggressive treatment strategies with clearing of the future liver remnant by percutaneous ablation or atypical resection for patients with bilobar colorectal liver metastasis (Hitpass et al. 2020). The two-stage hepatectomy is indicated for bilateral, multinodular disease that is not amenable to complete removal by single hepatectomy. In the first stage, the less-invaded liver lobe is cleaned of its metastases in combination with contralateral PVE to induce hypertrophy of the future liver remnant. In the second stage, the tumor‐bearing liver lobe (deportalized liver lobe) is anatomically removed (Imai et al. 2021).

Associating liver partition and portal vein ligation for staged hepatectomy (ALPPS) was introduced in 2007 in Regensburg (Germany) as a procedure to induce a more rapid hypertrophy of the functional liver remnant. Briefly, ALPPS involves portal vein ligation of the to-be resected segments and in-situ split of the liver during the first stage. The rapid hypertrophic response allows removal of the deportalized liver in the second stage when volume and/or function of the future remnant liver is sufficient, usually after 1–2 weeks (Schlitt et al. 2017). The high morbidity and mortality rate (respectively, 68% and 12%) that was first described decreased to 27% and 9%, with increasing of the surgical experience (Schadde et al. 2014). Moreover, the introduction of the “partial ALPPS” that consist in preserving the middle hepatic vein and the transection of only 50–80% of liver parenchyma by the first operation lead to a significant reduction in perioperative morbidity (Linecker et al. 2017). The oncological effect of ALPPS for CLM was first described in a cohort study of 510 patients from 22 international centers. The 3 and 5 year cancer-specific survival after ALPPS were 59%, and 33%. The response to neoadjuvant chemotherapy was the strongest independent predictor of short- and long-term oncological outcome. T4 stage, right-sided localization of the primary colon tumor, and K/N-RAS mutation were negative predictor factors. Surprisingly, size of liver metastases, bilobar disease, or even concomitant lung metastases had no significant impact on oncological outcome after ALPPS (Petrowsky et al. 2020).

### Minimally invasive surgery

Minimally invasive surgery is increasingly being performed on the liver. The OSLO-COMET study was the first randomized controlled trial that compared laparoscopic vs open liver resection for CLM. It demonstrated that laparoscopic liver resection was associated with lower post-operative complication rates and shorter post-operative hospital stay as compared to open liver resection, with no differences in blood loss, operation time, or 90 day mortality rates (Fretland et al. 2018). A recent review of Kabir et al*.* showed the results of nine retrospective studies: they agreed that laparoscopic liver resection is associated with lower blood loss and blood transfusion rates in addition to shorter length of stay and reduced post-operative complications, with similar mortality rates to open surgery (Kabir et al. 2020). Robot-based liver surgery is considered to be a further development of laparoscopic liver surgery due to its three-dimensional display, the high range of motion of the instruments, the reduction in physiological tremors, and the option of microsurgical anastomoses. This technique allows a well exposition of the liver, so that tumour location is no longer a limit for mini-invasive surgery. Moreover, robotic surgery proved to be at least comparable with laparoscopic surgery in term of short- and long-term outcomes (Rahimli et al. 2020).

### Local ablation strategy

For patients with unresectable disease or significant comorbidities precluding resection, there are several alternative therapies able to spare liver parenchyma. The thermal ablation of tumours utilizes image guidance to deliver extreme temperatures to a tumour and its surrounding tissue. The advantages include its adaptability to minimally invasive approaches, the ability to spare liver parenchyma, and a low morbidity rate. Thermal ablation can be performed percutaneously, laparoscopically, or at laparotomy (Abdalla et al. 2013). Radiofrequency ablation (RFA) is the most commonly used form of thermal ablation in the treatment of liver tumours. In RFA, needles placed in and around the tumours deliver alternating electrical current in the radiofrequency range that generates heat. The limitation is that it is generally ineffective in tumours bigger than 3 cm, the rate of local recurrence is high, and there is a lack of pathologic evidence that the target lesion was completely ablated with sufficient tumor-negative margins. In addition, the heat generated by RFA can injure adjacent structures (Abdalla et al. 2013).

A further technique is the cryoablation that involves liquid nitrogen or argon gas being delivered into the liver tumour, guided by ultrasound. Ice crystal formation during rapid freezing causes destruction of cellular structure and kills the tumour cells. Cryoablation has fallen out of favor because of a higher complication rate and recurrence rate in comparison with RFA (Clark and Smith 2014).

Percutaneous ethanol injection (PEI) is a local treatment based on the chemical properties of alcohol. Intratumoural injection of highly concentrated ethanol induces protein denaturation, microvascular thrombosis, cellular dehydration, and coagulative necrosis of the tumor. Comparatively to physical ablation techniques, PEI shows several limitations. First, the requirement to repeat multiple PEI procedures may not be tolerated as well. Second, intra-tumoural ethanol diffusion is less predictable than thermal ablation. Moreover, this method has high tumoural recurrence rates and worse disease-free and overall survival rates than RFA (Revel-Mouroz et al. 2017).

CLM have been shown to depend heavily on the hepatic artery for most of their blood supply, whereas the normal liver parenchyma relies mainly on portal blood flow. Based on this concept, several techniques have been developed such as hepatic arterial infusion chemotherapy (HAI), transarterial chemoembolization (TACE), and selective internal radiation therapy (SIRT).

HAI is a technique that introduce chemotherapy directly through a catheter placed in the hepatic artery. A high concentration of antimetabolite substance reaches the tumour permitting a lower systemic toxicity. Floxuridine is the most commonly used chemotherapy (Abdalla et al. 2013). A study of Ammori et al. reached a 25% of conversion to complete resection with a combination of HAI and systemic chemotherapy. Moreover, 5 year survival was significantly better in the conversion group (Ammori et al. 2013).

TACE is the administration of embolic particles mixed with chemotherapeutic drugs. It produces a shutdown of blood flow and the simultaneous release of high doses of the drug. The most used chemotherapy is irinotecan (DEBIRI) (Clark and Smith 2014). A comparison of DEBIRI *versus* systemic therapy showed that DEBIRI prolonged the median survival and was associated with a greater tumour response in the liver. The regional approach was not inferior to FOLFIRI in preventing extrahepatic metastatic progression (Fiorentini et al. 2012).

In the SIRT procedure, a single dose of 2.0–3.0 Gbq of yttrium^90^ microspheres is delivered into the hepatic artery that results in selective tumour uptake and radiation (Khatri et al. 2007). The SIRFLOX study is the largest phase-III randomized controlled trial to assess the efficacy and safety of chemotherapy plus/minus SIRT in patients with metastatic colorectal cancer. The results failed to show an improvement in median progression-free survival and OS with the addition of SIRT. On the other hand, overall response rate in the liver was improved by the addition of SIRT (van Hazel et al. 2016; Wasan et al. 2017).

### Resection vs. RFA

RFA is predominantly used for patients with surgery-prohibitive comorbidities or with poor functional hepatic reserve after resection. Ruers et al*.* demonstrated that ablation gives an overall- and progression-free survival benefit in addition to chemotherapy if compared to chemotherapy alone (Ruers et al. 2012, 2015). Since ablation strategy has low complications rate, is less invasive, can be repeated to treat progression or new metastases, and does not require prolonged interruption of chemotherapy, there is a growing interest whether the RFA could reach same oncologic results as surgery in the treatment of small SLM. In the past years, multiple studies compared surgery to RFA for the treatment of SLM (Oshowo et al. 2003; Aloia et al. 2006; Lee et al. 2008; Hur et al. 2009; Kim et al. 2011; Aliyev et al. 2013) (Table 1). They all are retrospective works that compared liver resection *versus* local ablation: the last one was applied in case of

-Patient comorbidities that did not allow major surgery,
-Not sufficient functional liver remnant after surgery (cirrhosis or steatohepatitis),
-Ill located metastasis that did not allow a resection, or
-Patient decision.

**Table 1:** Resection versus RFA: OS, DFS, and local recurrence rate after treatment of SLM

References		Patients no	3 year OS (%)	*p*	5 year OS (%)	*p*	3 year DFS (%)	*p*	5 year DFS (%)	*p*	Local recurrence (%)	*p*
Oshowo et al. (2003)	RFA	25	52.6	n.s	–	–	–	–	–	–	–	–
	Resection	20	55.4		–		–		–		–	
Aloia et al. (2006)	RFA	30	57	** < 0.001**	27	** < 0.001**			0	**0.001**	37	** < 0.001**
	Resection	150	79		71				50		5	
Lee et al. (2008)	RFA	37	–	–	48.5	n.s	32.2	n.s	25.7	n.s	29.7	** < 0.001**
	Resection	116	–		65.7		34.5		30.1		6.9	
Hur et al. (2009)	RFA	25	60	**0.026**	25.5	**0.026**	–	–	–	–	28	n.s
	Resection	42	70		50.1		–		–		9.5	
Kim et al. (2011)	RFA	177	–	–	51.1	n.s	–	–	33.6	n.s	–	–
	Resection	278	–		51.2		–		31.6		–	
Aliyev et al. (2013)	RFA	44	81	n.s	57	n.s	32	n.s	–	–	18	**0.012**
	Resection	60	81		47		36		–		4	

Bold figures show statistically significant diffenences p< 0.05

RFA patients group received the ablation percutaneously or intraoperatively. The results about survival rates were not homogeneous: only two of six works showed a significant better OS in the surgical group and only the study of Aloia et al. demonstrated a significant better disease-free survival (DFS) for the surgical group (Aloia et al. 2006; Hur et al. 2009). The most studies agree that patient treated with RFA had significant higher local recurrence than surgical-treated patients (Aloia et al. 2006; Lee et al. 2008; Hur et al. 2009; Aliyev et al. 2013). Berber et al. analyzed the factors that influence the recurrence after RFA in a group of 1.032 tumours: tumour size, ablation margin, and blood vessel proximity demonstrate to be independent predictors for local recurrence. Due to blood vessel proximity, it is well known that hepatic blood flow leads to incomplete ablation with its cooling properties and is commonly termed ‘‘heat sink effect’’ (Pillai et al. 2015). Concerning the ablation margin, a margin of 10 mm all around the target tumour is the ideal result of any ablation. However, there is a discrepancy between the success of the ablation based on imaging and real microscopic status at the ablation margin (Petre and Sofocleous 2017). A study of Sotirchos et al. performed a biopsy of the ablation zone immediately after RFA and demonstrated that in 24% of the cases, viable tumour cells may be present within the ablation zone, even when postprocedural imaging displays sufficient ablation margins (Sotirchos et al. 2016).

Regarding the tumour size, two further studies emphasized the efficacy of RFA for metastasis smaller than 3 cm: in this subgroup of patients, the difference in OS, DFS, and local recurrence-free survival flattened out if compared with surgery (Kim et al. 2011; Hur et al. 2009). In a study of Aliyev et al. 44 laparoscopic RFA were performed and compared to 60 liver resections: the OS and DFS did not differ in the two groups; the local recurrence rate after RFA was higher than surgery even if it was one of the lowest reported (18%). Moreover, they reached a local tumour control rate of 82% for metastases smaller than 3 cm. Such results are not reproducible with percutaneous RFA, since the laparoscopic technique was guided by a laparoscopic ultrasonography device that allow to examine in detail the ablation margin and is able to detect additional liver tumour lesions not seen in the preoperative imaging (20% of all cases) (Aliyev et al. 2013). Since the results are very heterogeneous, two recent works performed a meta-analysis to confront local ablative techniques and surgery. Both study recognized a superiority in surgical resection in OS and DFS with reduced local recurrence, even in case of small (< 3 cm) resectable SLM (Di Martino et al. 2020; Gavriilidis et al. 2021).

Summing up, to date, there are no study that proved the superiority of RFA over surgery and liver resection remains the best oncological treatment for solitary liver metastasis. There is only an ongoing phase-III randomized trial comparing thermal ablation to liver resection for patients with small (< 3 cm) CLM (COLLISION trial) (Puijk et al. 2018). The results have not been yet published. Moreover, the LAVA trial was a multicenter randomized controlled trial that wanted to compare thermal ablation *versus* liver resection surgery in high surgical risk patients. It was stopped after 1 year of pilot study, because only 9 patients were randomized (Davidson et al. 2020).

### Prognostic factors

There is a general consensus that patients with SLM have a significant better prognosis than patients with multiple metastases (K.-M. Chan et al. 2014; Brouquet et al. 2011; Abdalla et al. 2004). A meta-analysis summarized information of survival after liver resection for CLM of papers published between 1999 and 2010. For SLM, a 5 year OS of 47.4% was reported, not so far from the result of multiple liver metastases (40.3%) (Kanas et al. 2012). To improve the reliability and applicability of the results, two recent works, one of which was developed from our study group, focused on a homogeneous group of patients characterized by SLM treated with curative intent surgery. Both studies recognized the presence of lymph-node metastasis as a strong negative prognostic factor, with worse OS and DFS such as higher rate of tumour recurrences. The size of metastasis, the T-category, and the site of the primary tumour (colon *versus* rectum) did not affect the survival rates (Shin et al. 2019; Acciuffi et al. 2018). There is a strong evidence that synchronous metastasis and presence of extrahepatic tumour are factors associated with worse prognosis (Lee et al. 2008; Acciuffi et al. 2018).

Moreover, right-sided primary colon tumor have worse survival rates if related to left-sided primary colon tumour (Benedix et al. 2010). Right-sided colon tumour shows more often activating mutations of RAS, BRAF, and PIK3CA genes. In contrast, left-sided colon tumour is characterized by a more frequent occurrence of chromosomal instability and a gene expression profile corresponding to an activation of the epithelial growth factor receptor (EGFR) pathway. Patients with RAS wild-type left-sided colon tumour had a markedly greater benefit from anti-EGFR-based therapy (Holch et al. 2017).

For solitary liver metastasis, the size of the liver resection does not influence the post-operative outcome in case an R0 status has been reached (Acciuffi et al. 2018). The importance of the microscopic status of the resection margin is largely described and is widely accepted that a microscopically positive (R1) margin is strongly correlated with worse OS (Altendorf-Hofmann and Scheele 2003; Pawlik et al. 2005; Poultsides et al. 2010; Tranchart et al. 2013). Since the 1980s, there has been a general consensus that the optimal surgical margin during resection of CLM should measure ≥ 1 cm. In fact, some authors even suggested that inability to accommodate a 1 cm margin should perhaps preclude a patient from being considered for hepatic resection (Cady and Stone 1991; Ekberg et al. 1986). More recently, surgeons have progressively moved from the “1 cm” rule to the “1 mm” rule, and multiple reports have demonstrated that margin width does not affect the outcome as long as a negative margin is achieved (Altendorf-Hofmann and Scheele 2003; Poultsides et al. 2010; Viganò et al. 2018). This aspect increases the responsibility of the surgeon who should strive assiduously to achieve complete macro- and microscopic resection of CLM to help ensure the best outcomes for the patient. Actually, there is an additional distinction that should be done between standard R1 parenchymal resection (tumour exposure along the parenchymal transection line) and R1 vascular resection (detachment of CLM from major intrahepatic vessels). Although both are types of R1 resection, a recent study demonstrated that R1 vascular resection guaranteed exactly the same local control as R0 resection, confirming the hypothesis that vessels in contact with CLM limit tumor spread (Viganò et al. 2016). The role of adjuvant chemotherapy after resection of CLM has not been yet defined. Several trials that try to find an answer failed to reach trustable results due to poor recruitment, early termination, and usage of non-modern chemotherapy by today’s standards (G. Chan and Chee 2020). The most recent trial JCOG0603 compared 6 months of post-operative mFOLFOX6 with observation alone. The chemotherapy arm had a significant better 3 year DFS. However, the 5 year OS was 83.0% in the control arm compared to 69.5% for patients receiving adjuvant chemotherapy (Kanemitsu et al. 2021).

### Solitary metastasis: “the only one” or “the first one”?

The incidence of early recurrence that has been reported in the literature is moderately high. In a LiverMetSurvey-Based Study of 6,025 patients, 2,734 (45%) had recurrence and 23.4% of these one appeared in the first 6 months after surgery (Viganò et al. 2013). In our previous work, about half of the recurrences appeared in the first 12 months after resection (Acciuffi et al. 2018). Multiple works showed that early recurrence is highly associated with worse disease-specific survival (Tan et al. 2013; Viganò et al. 2021). A recent work of Fromer et al. showed that in a group of 259 major liver resections for CLM, 12.6% had a recurrence event within 6 months after operation and experienced a futile liver resection with significantly lower OS (Fromer et al. 2021). We can suppose that patients with early recurrence probably had subclinical undetected liver lesions at the time of surgery and thus small that they could not be identified even with the intraoperative ultrasound (occult metastases). In these cases, the surgeon did not resect the “only one” metastasis but rather the first detectable lesion of multiple metastases. A previous study of Yokoyama et al. demonstrated that immunohistochemically detected hepatic micrometastases were found in about a half of patients treated with curative liver resection. The presence of micrometastases was a predictive factor for increased risk of intrahepatic recurrence and was a poor prognostic indicator of survival (Yokoyama et al. 2002). Since the modern diagnostic tools are not able to identify the micrometastases yet, only the time factor permits to evaluate the aggressiveness of the tumour and discern patients with fast tumour progression. To recognize among the SLM the one with worse biology, a “test-of-time” strategy for those patients with poor prognostic indicator could be taken into consideration. If after a few months, a re-stage of the patients reveals new metastases, it can be assumed that they were present as subclinical undetected micrometastases at the time of diagnosis. After the reevaluation, the strategy of treatment has to be discussed again to reach a complete surgical resection of the tumour or, in case of unresectable disease, another treatment strategy should be adopted such as liver-parenchyma-sparing surgical technique, local ablation, or systemic therapy or a combination of the previous. Lambert et al. delayed resection for 3–6 months in 42 of 73 total patient with liver metastases, to allow occult disease to become clinically detectable and spare the morbidity and mortality of major surgery in those patients that become unresectable during the interval to reevaluation. There was no significant difference in survival between those patients who underwent immediate hepatic resection and those who underwent interval reevaluation. In the reevaluation group, 28% of the patient were spared the morbidity of noncurative surgery (Lambert et al. 2000). A meta-analysis of 2880 patients with synchronous metastases proved that simultaneous resection was as efficient as a delayed procedure for the long-term oncological outcomes, since there were no significant differences of both OS and DFS, confirming that the delayed procedure is safe and feasible (Yin et al. 2013). An important concern is the risk of “missed opportunities” through loss of resectability because of increased size of the initial metastasis: patients who present a metastasis with an unusual location, such that any further growth might technically preclude resection, immediate removal should be preferred.

## Conclusion

Surgery remains the first choice of treatment for solitary colorectal liver metastases. Nowadays, the surgical skills and the modern multidisciplinary hypertrophy strategy lead an increased number of patients to be eligible for surgery. In case of unresectable disease, multiple local ablation treatments should be considered in combination to chemotherapy. Trustable negative predictive factors that can help in the interdisciplinary treatment decision are N-category of the primary tumor, synchronous metastasis, extrahepatic tumour location, right-sided primary colon tumor, and R1 status. A test-of-time is a possibility for high selected patients to reveal subclinical metastases permitting to treat all the tumour manifestations or to offer an alternative strategy of therapy.

## Data Availability

Not applicable.
